# An initial report of memory avoidance whole brain radiotherapy to treat brain metastases

**DOI:** 10.1093/noajnl/vdag211

**Published:** 2026-08-13

**Authors:** Joshua D Palmer, Erica L Dawson, Yilun Sun, Jiawei Yu, Khaled Dibs, Alex R Ritter, Daniel Boulter, Ansel P Nalin, Raj Singh, Sasha J Beyer, Simeng Zhu, Dukagjin M Blakaj, John C Grecula, Raju R Raval, Shayla Vazquez, Samantha Whitman, Carolyn J Presley, Clement Pillainayagam, Pierre Giglio, Evan M Thomas, Haley K Perlow

**Affiliations:** Department of Radiation Oncology, Ohio State University Wexner Medical Center, Columbus, Ohio, USA; Department of Neurology, Ohio State University Wexner Medical Center, Columbus, Ohio, USA; Department of Population and Quantitative Health Sciences, Case Western Reserve University, Cleveland, Ohio, USA; Department of Population and Quantitative Health Sciences, Case Western Reserve University, Cleveland, Ohio, USA; Department of Radiation Oncology, Ohio State University Wexner Medical Center, Columbus, Ohio, USA; Department of Radiation Oncology, Ohio State University Wexner Medical Center, Columbus, Ohio, USA; Department of Radiology, Ohio State University Wexner Medical Center, Columbus, Ohio, USA; Department of Radiation Oncology, The University of Texas MD Anderson Cancer Center, Houston, Texas, USA; Department of Radiation Oncology, Ohio State University Wexner Medical Center, Columbus, Ohio, USA; Baptist Health South Florida, Lynn Cancer Institute, Boca Raton, Florida, USA; Department of Radiation Oncology, Ohio State University Wexner Medical Center, Columbus, Ohio, USA; Department of Radiation Oncology, Ohio State University Wexner Medical Center, Columbus, Ohio, USA; Department of Radiation Oncology, Ohio State University Wexner Medical Center, Columbus, Ohio, USA; Department of Radiation Oncology, Ohio State University Wexner Medical Center, Columbus, Ohio, USA; Department of Radiation Oncology, Ohio State University Wexner Medical Center, Columbus, Ohio, USA; Department of Neurology, Ohio State University Wexner Medical Center, Columbus, Ohio, USA; Department of Neurology, Ohio State University Wexner Medical Center, Columbus, Ohio, USA; Department of Medical Oncology, Ohio State University Wexner Medical Center, Columbus, Ohio, USA; Department of Solid Tumor Oncology, University Hospitals Seidman Cancer Center, Case Western Reserve University, Cleveland, Ohio, USA; Department of Neurology, Ohio State University Wexner Medical Center, Columbus, Ohio, USA; Department of Radiation Oncology, University Hospitals Seidman Cancer Center, Case Western Reserve University, Cleveland, Ohio, USA

**Keywords:** brain metastases, cognition, hippocampus, quality of life, radiation therapy

## Abstract

**Background:**

Hippocampal avoidance whole brain radiotherapy (HA-WBRT) with memantine is standard of care for patients with extensive brain metastases requiring radiation. However, other brain structures have critical memory and cognition roles, including the corpus callosum, fornix, amygdala, hypothalamus, and pituitary. A subset of patients enrolled on a Phase 2 Randomized Controlled Trial received an advanced “memory-avoidance” WBRT (MA-WBRT) approach that spared these substructures in addition to the hippocampus.

**Methods:**

Patients with >15 brain metastases were enrolled in the ATHENA Trial and received MA-WBRT. All patients received 30 Gy in 10 fractions of MA-WBRT and were prescribed memantine. Cognition was measured by Hopkins Verbal Learning Test-Revised, Controlled Oral Word Association Test, and Trail Making Test A/B, with cognitive decline defined as decline on at least one assessment using the reliable change index.

**Results:**

Between August 2022 and May 2024, 29 patients were prescribed MA-WBRT. Decline in neurocognitive function at 3 and 6 months for patients receiving MA-WBRT was 17.2% and 48.3%, respectively. There was only 1 failure in a memory avoidance substructure (occurring in the right fornix 10 months after enrollment), and this was associated with concurrent distant intracranial failure outside the memory avoidance zone.

**Conclusions:**

The cognitive decline rates of 17.2% and 48.3% at 3 and 6 months for patients receiving MA-WBRT compare favorably to the 3- and 6-month cognitive decline rates of 50% and 60% seen on NRG CC001. A direct comparison of MA-WBRT plus memantine vs. HA-WBRT plus memantine is forthcoming in a randomized phase 2 trial (NCT07248228).

Key Points Herein, we report the final outcomes of patients in a phase 2 trial who received MA-WBRT.

Our analyses show improvements in the incidence and rate of cognitive decline compared to historical data without isolated failures within the avoidance structures.

Importance of the Study

This is the first publication of the clinical and cognitive outcomes of patients receiving MA-WBRT for brain metastases. There are no other known WBRT clinical trials sparing additional cognitive substructures in addition to the hippocampus. All patients received 30 Gy in 10 fractions with avoidance of the hippocampus, amygdala, corpus callosum, fornix, hypothalamus, and pituitary. The final analyses show relative improvements in the incidence and rate of cognitive decline compared to historical NRG CC001 data. Notably, there were no isolated failures necessitating additional radiation treatment within the avoidance structures, confirming the safety of MA-WBRT. These data posit additional benefit for avoidance of additional cognitive organs at risk for patients with brain metastasis receiving HA-WBRT and memantine.

Brain metastases are the most common intracranial malignancy and often require treatment with multi-modality therapy. As treatment advances have improved survival, neurocognitive functioning and quality of life have become critical endpoints in this patient population.^1^^,^^2^ For patients with limited brain metastases, stereotactic radiosurgery (SRS) is the treatment of choice given the effects of whole brain radiotherapy (WBRT) on verbal learning and delayed recall.^3-5^ However, for patients with extensive brain metastases, or those who cannot undergo SRS, WBRT remains a reasonable treatment option. For patients with a large number of brain metastases and ineligible for SRS, the standard of care involves hippocampal avoidance whole brain radiotherapy (HA-WBRT) with memantine.^6^ The hippocampus is the most consistently validated intracranial structure for its critical role in memory. Therefore, sparing of the hippocampus is associated with better cognitive outcomes compared to standard WBRT.^7^^,^^8^ To date, there have not been prospective studies validating the cognitive effects of sparing other intracranial structures in patients receiving WBRT.

For patients in whom WBRT is necessary, reducing radiation dose to critical cognitive substructures can mitigate the cognitive decline seen in historical WBRT and HA-WBRT trials. Additional cognitive substructures with a documented relationship between radiation dose and function include the following: the amygdala, a key limbic structure involved in emotional and social processes^9^^,^^10^; the corpus callosum, a substructure that assists with processing speed, attention, and executive function^11-13^; the fornix, an efferent tract of the hippocampus associated with thinking and memory^14^^,^^15^; and the hypothalamic-pituitary axis which impacts memory and fatigue.^16^ An advanced volumetric modulated arc therapy technique termed memory avoidance whole brain radiotherapy (MA-WBRT) was therefore developed to avoid these critical cognitive substructures.^17^ These substructures have a low propensity for brain metastases, and it was hypothesized that these structures could be safely avoided without significantly increasing the risk of intracranial relapse.^18^

Herein, we report the final cognitive outcomes of a subset of patients in a phase 2 trial who received MA-WBRT. We hypothesize that MA-WBRT cognitive outcomes compare favorably to HA-WBRT outcomes without increasing the risk of intracranial recurrence and retreatment.

## Methods

### Study Design

The ATHENA Trial (ClinicalTrials.gov identifier: NCT05503251) is an American, single-center, phase 2 randomized controlled trial including patients with brain metastases planned for radiotherapy treatment. Patients were stratified by radiation cohort (>15 versus ≤15 brain metastases) and performance status. They were randomized 1:1 to either the control arm or the experimental neuropsychological integration arm involving elements of cognitive rehabilitation. The investigators obtained informed consent from each participant, and the human investigations were performed after approval by a local Human Investigations Committee. Pertinent inclusion criteria included a stage IV diagnosis of malignancy with brain metastases, planned for treatment with radiotherapy, primarily English-speaking, and with an estimated survival of ≥6 months. Pertinent exclusion criteria included prior whole brain radiotherapy or preexisting neurologic condition (multiple sclerosis, dementia, or mental disability). It was previously reported that there was no difference in neurocognition between the control and intervention arms.^19^ All patients with >15 brain metastases received MA-WBRT. All MA-WBRT patients were contoured and planned using published guidelines, including use of previously used hippocampus dose constraints for additional organs at risk.^17^ Baseline and treatment-related characteristics were collected for all patients enrolled on the study. The primary endpoint in MA-WBRT-specific analyses was occurrence of cognitive decline as measured by reliable change index scores identified for each cognitive measure.

Before treatment, all study patients completed the Functional Assessment of Cancer Therapy—Brain (FACT-Br), European Organization for the Research and Treatment of Cancer Quality of Life Questionnaire (EORTC QLQ-30), General Anxiety Disorder-7 (GAD-7), Patient Health Questionnaire-9 (PHQ-9), and Patient-Reported Outcomes Measurement Information System-8 (PROMIS-8) to assess quality of life, severity of anxiety and depression symptoms, and general symptoms and functioning. Hopkins Verbal Learning Test-Revised (HVLT-R), Controlled Oral Word Association Test (COWAT), and Trail Making Test A/B (TMT A/B) were administered by trained research assistants to evaluate verbal learning and memory, verbal fluency, and executive function, respectively, and were the measures used to determine cognitive decline. These measures were used to evaluate cognitive decline. These tests were repeated at 3, 6, 9, and 12 months after enrollment for all participants.

### Statistics

All quality of life (QOL) and cognitive testing results were converted to normative scores prior to analysis. For overall survival, the survival rates for each treatment group were estimated using the Kaplan-Meier method and were compared using the Log-Rank test. Neurocognitive decline was defined as a decline in at least one assessment using test-specific reliable change index scores.^8^ When analyzing neurocognitive decline as an event of interest, deaths without decline were considered a competing risk event. Cumulative incidence rates of neurocognitive decline were estimated and were then compared using Gray’s test. For all statistical analyses, a *P*-value <.05 was defined as statistically significant.

## Results

From August 2022 to May 2024, 29 patients were planned for MA-WBRT (Table 1). A total of 26 patients initiated and completed radiation. The median age was 64 years (IQR 54, 69). About 64% of patients were female. About 48% of patients were diagnosed with primary lung cancer. Most patients (72.4%) had a Karnofsky Performance Status (KPS) of 80 or higher. The median number of metastases was 29 (IQR 17, 41). The median tumor volume was 3.5 ccs (IQR 1.3, 17.2). Four (13.8%) patients had brainstem metastases. About 37.9% of patients had controlled systemic disease, and most patients (79%) had concurrent extracranial metastases.

**Table 1.** Patient characteristics

Characteristic	Overall (*N* = 29)^a^
Age	64.0 (54.0, 69.0)
KPS	
60	4 (13.8)
70	4 (13.8)
80	8 (27.6)
90	10 (34.5)
100	3 (10.3)
Education	
Graduate school	1 (3.4)
Some/completed college	13 (44.8)
High school or less	11 (37.9)
Unknown	4 (9.1)
Gender	
Female	18 (62.1)
Male	11 (37.9)
Race	
Black or African American	3 (10.3)
White	26 (89.7)
Primary tumor histology	
Breast	7 (24.1)
Genitourinary	4 (13.8)
Head and neck	1 (3.4)
Thoracic	14 (48.3)
Melanoma	2 (6.9)
Other	1 (3.4)
Extracranial disease control	
Controlled	11 (37.9)
Uncontrolled	18 (62.1)
Extracranial metastases	
Absent	6 (20.7)
Present	23 (79.3)
Number of brain metastases	29 (17, 41)
Tumor volume (ccs)	3.5 (1.3, 17.2)
Previous brain radiation	
No	21 (72.4)
Yes	8 (24.5)

a Median (Q1, Q3); *n* (%). KPS, Karnofsky performance status; Ccs, cubic centimeters.

All patients received memantine. All patients received 30 Gy in 10 fractions, with 12 patients (41.4%) having a gross tumor volume (GTV) boost to 35 Gy. There were no patients who required surgical resection of a metastasis immediately before or after treatment.

**Figure 1.**
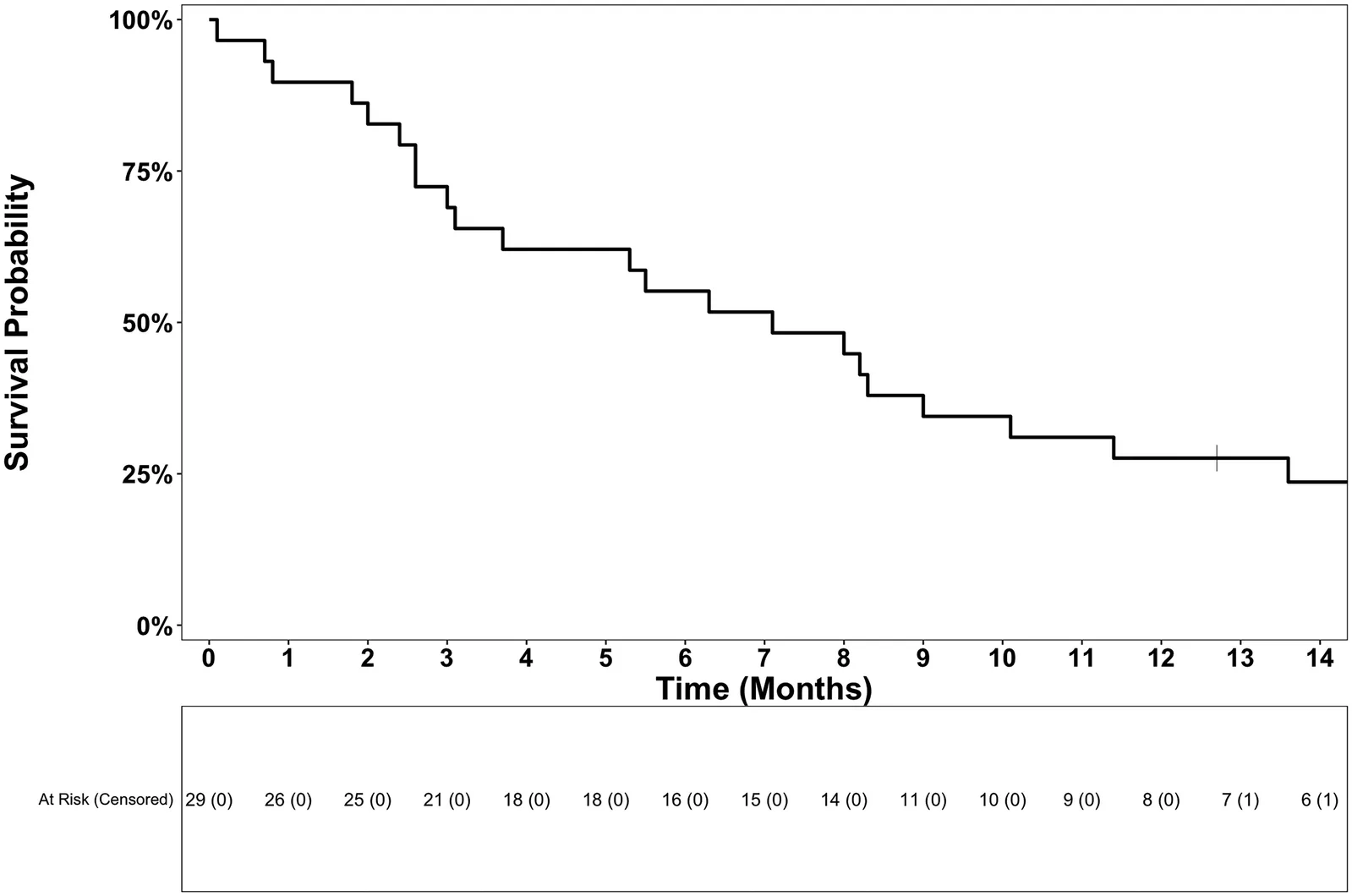
Overall survival probability.

**Figure 2.**
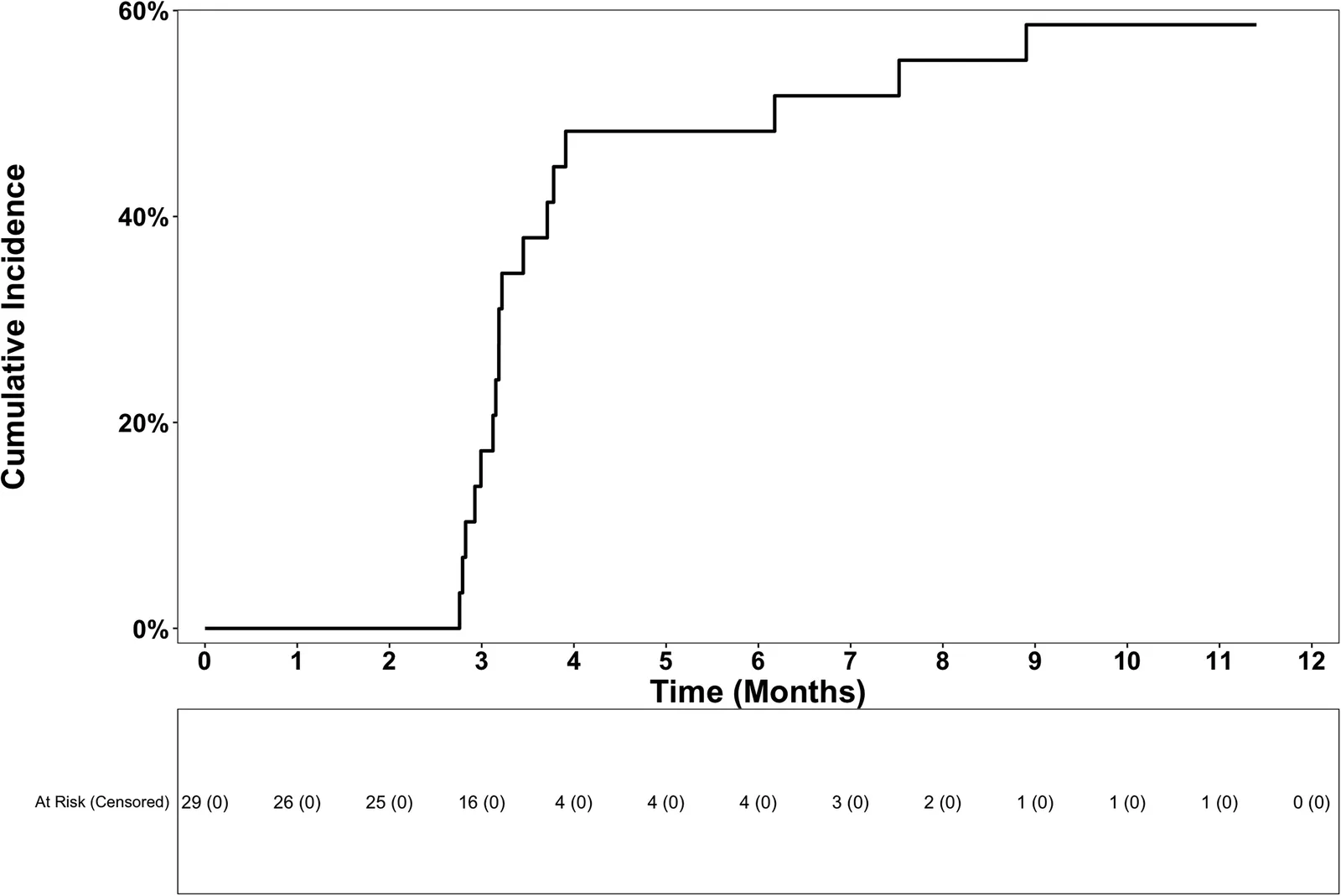
Cumulative incidence of cognitive decline.

The median overall survival or time to last follow-up was 8.1 months, with 27.6% of patients living at least 1 year (Figure 1). The incidence of cognitive decline at 3, 6, 9, and 12 months was 17.2%, 48.3%, 58.6%, and 58.6%, respectively (Figure 2). Overall, the most common indicator of cognitive decline at 3 months was seen in the HVLT-R Total Recall, with 8 patients showing decline from baseline testing to 3 months (Table 2). Only 2 patients experienced a decline in TMT A from baseline testing to 3 months. Overall, 12 patients did not have full cognitive testing after their baseline evaluation.

**Table 2.** Incidence of cognitive decline on individual tests at 3 months

Test	Number of patients
HVLT-R total recall	
No decline	9 (52.9)
Decline	8 (47.1)
No follow up testing	11
HVLT-R delayed recall	
No decline	10 (58.8)
Decline	7 (41.2)
No follow-up testing	11
HVLT-R delayed recognition	
No decline	13 (76.5)
Decline	4 (23.5)
No follow-up testing	11
TMT part A	
No decline	14 (87.5)
Decline	2 (12.5)
No follow up testing	12
TMT part B	
No decline	12 (75.0)
Decline	4 (25.0)
No follow-up testing	12
COWAT	
No decline	12 (75.0)
Decline	4 (25.0)
No follow-up testing	12

HVLT-R, Hopkins verbal learning test; TMT, trail making test; COWAT, controlled oral word association test.

**Figure 3.**
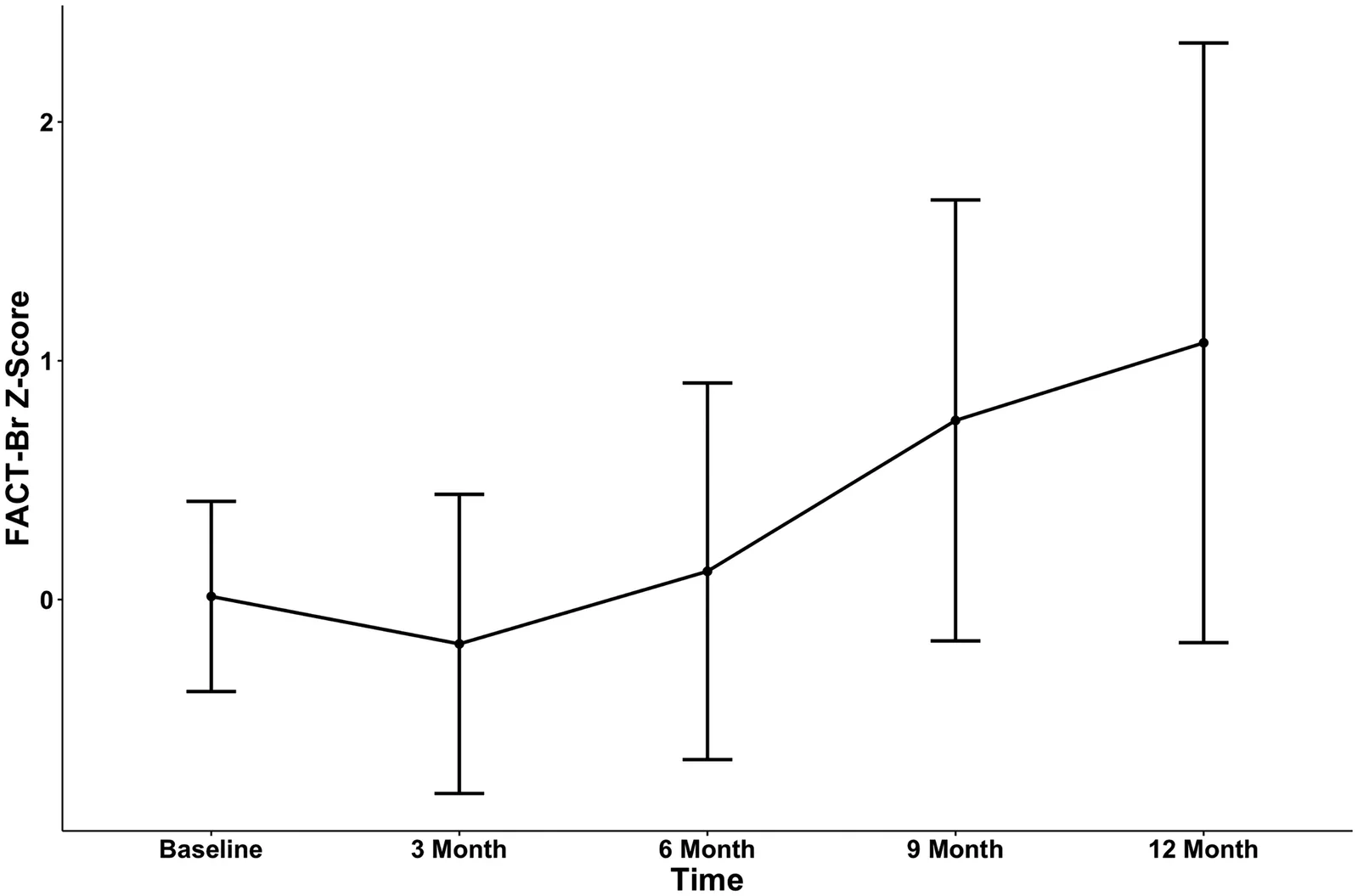
Changes in FACT-Br over time.

There was a trend toward improvement in most quality of life (QOL) metrics, including Fact-Br (Figure 3), Fact-G (Supplementary Table S1), Fact-Br Cognitive Index (Supplementary Table S2),^20^ EORTC indices (Supplementary Tables S3-S6), and PROMIS-8 (Supplementary Table S7). The PHQ-9 and GAD-7 showed improvement in symptoms of depression and anxiety over time (Supplementary Tables S8 and S9).

Overall, 9 patients experienced CNS progression after MA-WBRT. There were 7 patients who developed distant progression and one patient who developed local progression. One patient experienced both distant and local progression events (Table 3). Of the 2 patients with local progression, 1 patient received a GTV boost and 1 did not. There was only 1 failure in a memory avoidance substructure, which occurred in the right fornix 10 months after enrollment. This event was associated with a concurrent distant intracranial failure outside the memory avoidance zone. One patient developed classical leptomeningeal disease 19 months after diagnosis.

**Table 3.** Adverse events

Event	Number of patients
CNS progression	
No	20 (69.0)
Yes	9 (31.0)
Distant progression	
No	21 (65.5)
Yes	8 (27.6)
Local progression	
No	27 (93.1)
Yes	2 (6.9)
Cognitive substructure failure	
No	28 (96.6)
Yes	1 (3.4)
Isolated cognitive substructure failure	
No	29 (100.0)
Yes	0 (0)
Leptomeningeal disease	
No	28 (96.6)
Yes	1 (3.4)

## Discussion

This is the first publication of the clinical and cognitive outcomes of patients receiving MA-WBRT for brain metastases. All patients received 30 Gy in 10 fractions with avoidance of the hippocampus, amygdala, corpus callosum, fornix, hypothalamus, and pituitary. The final analyses show relative improvements in the incidence and rate of cognitive decline compared to historical NRG CC001 data.^8^ These data posit additional benefit for avoidance of additional cognitive organs at risk for patients with brain metastasis receiving HA-WBRT and memantine.

The rationale of avoiding radiation dose to additional cognitive organs at risk is well supported. The dose-dependent relationships between the hippocampus and cognitive function are already well established for both benign/low grade tumors and metastatic brain tumors.^7^^,^^8^^,^^21^ Injury to the corpus callosum and intrahemispheric white matter has been associated with attention deficits and a decline in processing speed after radiation.^11^ Decreased memory formation and impaired recall are closely associated with injury to the fornix.^15^ Mean radiation dose to the amygdala is associated with volume atrophy, which may impact emotional processing and memory.^9^ Hypothalamic-pituitary axis dysfunction after brain radiotherapy has been linked to declines in quality of life and cognition.^16^^,^^22^ These data support the hypothesis that radiation-induced cognitive decline affects multiple domains such as memory, attention, processing speed, and social cognition. These effects likely reflect injury to a distributed neural network involving multiple cognitive substructures, not just the hippocampus. Considering this, MA-WBRT may be a safe and effective approach to mitigate those effects.

NRG-CC001 provides level 1 evidence establishing the benefit of avoiding the hippocampus and prescribing memantine for patients with extensive brain metastases receiving WBRT.^8^ The risk of cognitive failure was significantly lower (HR 0.76) in the HA-WBRT plus memantine arm versus the WBRT plus memantine arm, with 3- and 6-month cognitive failure rates of approximately 50%-60% in the HA-WBRT arm and 60%-70% in the WBRT arm. Although not powered for a direct comparison, our MA-WBRT cognitive failure data, using the same tests and definitions, showed a cognitive decline rate of 17% and 48% at three and six months, respectively. NRG CC001 also showed improvement in symptom interference and cognitive symptoms with HA-WBRT over time. Quality of life and symptom burden are difficult to compare between the MA-WBRT ATHENA Trial patients and NRG CC001 patients because different measures were used. MA-WBRT generally had quality of life and symptoms of anxiety/depression improvements over time, although that may be attributed to selection bias of long-term survivors. Our data suggest the added utility of avoiding additional cognitive substructures in this patient population and support a randomized trial comparing MA-WBRT with HA-WBRT.

The risk of recurrence associated with volumetric de-escalation is an important consideration for patients receiving MA-WBRT. Gondi et al^23^ showed that metastases within 5 mm of the hippocampus were observed in 8.6% of patients and overall comprised 3.0% of brain metastases. A systematic review of 5,374 patients with over 32,570 brain metastases showed that 4.4% and 9.2% of brain metastasis patients had hippocampal and perihippocampal brain metastases, respectively.^24^ In NRG CC001, there were paradoxically more relapses (16 versus 11) in the WBRT plus memantine arm compared to the HA-WBRT plus memantine arm. In the ATHENA MA-WBRT cohort, metastases were allowed within 5 mm of the at-risk cognitive substructures but were required to be fully covered by the prescription dose at the expense of cognitive substructure constraints.^17^ Notably, there were no isolated failures necessitating additional radiation treatment within the avoidance structures, confirming the safety of MA-WBRT.

One strength of this study is validation of a novel cognitive substructure sparing WBRT technique with prospectively collected cognitive and quality of life data. There are no other known WBRT clinical trials sparing additional cognitive substructures in addition to the hippocampus. MA-WBRT may be safe and effective in reducing the cognitive decline seen with historical WBRT or HA-WBRT data. One major limitation is sample size; a large, randomized trial is needed to confirm the benefit of MA-WBRT. Standard hippocampal constraints were used for the additional cognitive substructures, but each structure may have different radiosensitivity or dose thresholds necessary to minimize cognitive decline. Competing events such as baseline intracranial disease burden, intracranial progression, systemic progression, and cancer-directed therapies may also impact quality of life and cognition. Cognitive recovery was not evaluated due to small sample size and aforementioned competing events.^25^ Death from systemic disease may bias cognitive data toward healthier survivors. Endocrine function was not serially assessed for patients on study. Clinical trial neurocognitive testing included a standard battery used in many radiation oncology trials, including RTOG 0614 and NRG CC001,^8^^,^^26^ but more robust cognitive testing may identify nuanced changes that better quantify the effect on individual neurocognitive substructures.

## Conclusion

A subset of patients on the Phase 2 ATHENA Trial received a novel radiotherapy technique termed MA-WBRT sparing the hippocampus, amygdala, corpus callosum, fornix, hypothalamus, and pituitary. MA-WBRT may lead to a reduction in neurocognitive decline compared to historical data of patients enrolled in NRG CC001 who received HA-WBRT. Additionally, MA-WBRT does not appear to increase the risk of intracranial failure. A direct comparison of MA-WBRT plus memantine vs. HA-WBRT plus memantine is forthcoming in a randomized phase 2 trial (NCT07248228).

## Supplementary Material

vdag211_Supplementary_Data

## Data Availability

Research data are stored in an institutional repository and will be shared upon request to the corresponding author.

## Author

Experimental Design (H.K.P., E.L.D., J.D.P.), Implementation of Project (H.K.P., E.L.D., Y.S., J.Y., K.D., A.R., D.B., A.P.N., R.S., S.J.B., E.M.T., S.Z., D.M.B., J.C.G., R.R.R., S.V., S.W., C.J.B., C.P., P.G., J.D.P.), Analysis and Interpretation of Data (H.K.P., E.L.D., Y.S., J.Y., K.D., A.R., D.B., A.P.N., R.S., S.J.B., E.M.T., S.Z., D.M.B., J.C.G., R.R.R., S.V., S.W., C.J.B., C.P., P.G., J.D.P.).
