# Supplementary material for: An initial report of memory avoidance whole brain radiotherapy to treat brain metastases
Source: Neurooncol Adv. 2026 Aug 13;8(1):vdag211. doi: 10.1093/noajnl/vdag211 (PMC13528770; doi:10.1093/noajnl/vdag211)
Supplement: Supplementary file 1 [file vdag211_supplementary_data.docx]

Supplemental Figure 1.

Supplemental Figure 2.

Supplemental Figure 3.

Supplemental Figure 4.

Supplemental Figure 5.

Supplemental Figure 6.

Supplemental Figure 7.

Supplemental Figure 8.

Supplemental Figure 9.

**Supplemental Figure Legends**

Supplemental Figure 1. Changes in Fact-G Over Time.

Supplemental Figure 2. Changes in Fact-Br Cognitive Index Over Time. Y Axis shows raw scores. Higher scores reflect better subjective cognitive complaints.

Supplemental Figure 3. Changes in EORTC Global Health/Quality of Life Over Time.

Supplemental Figure 4. Changes in EORTC Physicial Functioning Over Time.

Supplemental Figure 5. Changes in EORTC Emotional Functioning Over Time.

Supplemental Figure 6. Changes in EORTC Fatigue Over Time.

Supplemental Figure 7. Changes in PROMIS-8 Over Time.

Supplemental Figure 8. Changes in GAD-7 Over Time. GAD - General Anxiety Disorder.

Supplemental Figure 9. Changes in PHQ-9 Over Time.
